# Food intake rates of inactive fish are positively linked to boldness in three‐spined sticklebacks Gasterosteus aculeatus

**DOI:** 10.1111/jfb.12934

**Published:** 2016-03-04

**Authors:** J. W. Jolles, A. Manica, N. J. Boogert

**Affiliations:** ^1^ Department of ZoologyUniversity of CambridgeDowning StreetCambridge CB2 3EJU.K.

**Keywords:** animal personality, body size, energy, foraging, metabolism, pace‐of‐life

## Abstract

To investigate the link between personality and maximum food intake of inactive individuals, food‐deprived three‐spined sticklebacks Gasterosteus aculeatus at rest in their home compartments were provided with ad libitum prey items. Bolder individuals ate considerably more than shyer individuals, even after accounting for body size, while sociability did not have an effect. These findings support pace‐of‐life theory predicting that life‐history strategies are linked to boldness.

It is now well known that consistent individual differences in behaviour, referred to as animal personality, are ubiquitous across the animal kingdom (Réale *et al.*, 2007; Dingemanse & Wolf, 2010; Sih *et al.*, 2015). Personality differences have been shown to be linked to fitness, to affect population dynamics, and to have fundamental ecological and evolutionary implications (Réale *et al.*, 2007; Wolf *et al.*, 2007; Dingemanse & Wolf, 2010; Conrad *et al.*, 2011). The major question remains, however, why animal personalities exist in the first place.

One of the most prominent theories to explain animal personalities from an adaptive perspective is that they exist because of underlying individual differences in state (Dingemanse & Wolf, 2010; Sih *et al.*, 2015), with the most widely proposed mechanism explaining personality differences in the context of broad life‐history strategies (Stamps, 2007; Wolf *et al.*, 2007; Biro & Stamps, 2010), integrating behaviour into the concept of a pace‐of‐life syndrome (Réale *et al.*, 2010). Central to this theory is that differences in traits such as boldness and aggression may arise through growth–mortality trade‐offs (Stamps, 2007; Biro & Stamps, 2008), effectively linking energetics with animal personality research (Careau & Garland, 2012). According to this view, individuals with high rates of growth and fecundity are expected to show physiological and behavioural adaptations associated with greater energy needs, such as higher rates of food intake and a greater tendency to take risks (*i*.*e*. bolder), both as a cause and consequence of their fast lifestyle (Biro & Stamps, 2008; Careau & Garland, 2012).

Empirical evidence is accumulating to support this theory: traits such as activity, aggressiveness and boldness have been found to positively correlate with growth, fecundity and other life‐history traits (Biro & Stamps, 2008; Burton *et al.*, 2011; Conrad *et al.*, 2011; Careau & Garland, 2012), and are positively related to rates of food consumption (Biro & Stamps, 2008). For example, Ioannou *et al.* (2008) showed that pairs of three‐spined sticklebacks *Gasterosteus aculeatus* L. 1758 that were quicker to leave refuge took less time to explore a potentially risky environment and consumed more live prey than those that hid under cover for longer. In addition, individuals with higher growth rates and fecundity would also require higher‐capacity ‘metabolic engines’ (Biro & Stamps, 2010), which is reflected by their higher resting metabolic rates (RMR; Huntingford *et al.*, 2010; Burton *et al.*, 2011; Martins *et al.*, 2011). Therefore, even when not currently engaging in any energetically expensive activities, such individuals are predicted to have higher energy requirements and thus food intake (Biro & Stamps, 2010).

Here, for the first time, it is tested whether personality differences are linked to food intake rates when individuals are at rest and risk‐reward trade‐offs are kept at a minimum, providing a more mechanistic link between boldness and food intake compared to previous work focused on ecological consequences (Ioannou *et al.*, 2008). Most variation in the food intake of inactive individuals is expected to be due to body size, with larger individuals eating more (Beukema, 1968; Allen & Wootton, 1984). Nevertheless, as boldness has been shown to positively correlate with growth and fecundity (Biro & Stamps, 2008; Careau & Garland, 2012), and risk‐taking behaviour with RMR (Killen *et al.*, 2011), it was predicted that bolder individuals would have a higher maximum food intake than shyer individuals, even when at rest and after accounting for body size. In contrast, personality traits that may not be strongly linked to growth or fecundity, such as sociability, are expected to not affect maximum foraging rates when at rest.

To test these predictions, 96 *G*. *aculeatus* were randomly selected from a wild stock which had been caught in tributaries of the River Cam, Cambridge, U.K., and were socially housed in an environmentally controlled laboratory. During this time before the start of experiments (over 6 months), the socially kept *G*. *aculeatus* were fed bloodworms (*Chironomid* sp. larvae) *ad libitum* at the end of each day. Individuals were individually photographed to measure their standard length (*L*_S_, from tip of snout to caudal peduncle), which ranged from 3·06 to 5·25 cm (mean ± s.e. = 4·07 ± 0·04 cm). Mass (*M*) was estimated from total length (*L*_T_; mean ± s.e. = 5·13 ± 0·05 cm) based on *L*_T_ and *M* relationship data from *G*. *aculeatus* extracted from www.fishbase.org using the formula *M* = *a L*_T_ *^b^* (*a* = 0·0068, describes body shape and condition; *b* = 3·28, describes isometric growth in body proportions) following Froese *et al.* (2014). This formula thus does not take into account any individual variation in other body measurements. The resulting *M* estimates ranged from 0·56 to 3·02 g (mean ± s.e. = 1·50 ± 0·05 g). After photographing, individuals were solitary housed in compartments (18·5 cm × 9·5 cm; 18 cm deep) that were lined with gravel and contained an artificial plant for cover. To minimize stress of isolation, compartments had perforated transparent Perspex walls that enabled the transfer of visual and chemical cues of seven conspecifics in neighbouring compartments. Each compartment contained a 2 cm wide feeding dish at the plant cover so that individuals could feed while staying concealed under cover.

After 3 days of acclimatization, *G*. *aculeatus* were first assessed for boldness, *i*.*e*. their willingness to take risks, and sociability, *i*.*e*. their tendency to approach others excluding aggressive behaviour (Réale *et al.*, 2007). To quantify boldness, an experimental setup was used as detailed in Jolles *et al.* (2014, 2015). In short, individuals were placed in a rectangular tank (55 cm length × 15 cm width × 20 cm height) lined with sand in a slope ranging from a deep (15 cm × 10 cm; 13 cm depth), ‘safe’ area that contained an artificial plant for cover, to a shallow depth (3 cm) at the other side. Boldness was quantified as the amount of time an individual spent out of plant cover during the 30 min trial, with bolder individuals spending more time out of cover. To quantify sociability, individuals were placed in the larger middle compartment (30 cm width) of a tank (50 cm × 30 cm, 8 cm water depth) that was lengthwise divided by two transparent Perspex partitions. One of the two smaller side compartments (10 cm width) contained five conspecifics. Sociability was quantified by measuring the average distance of the focal individual from the compartment containing the conspecific shoal during a 15 min trial. The conspecific shoal was created by randomly selecting individuals from the stock tanks, and allowed to acclimatize to the compartment for 45 min at the start of each test day. The position of the compartment housing the shoal was randomized every four trials, and after each compartment swap the shoal was allowed to acclimatize for 10 min before the start of the next trial. Eight individuals were tested in identical tanks simultaneously, and different conspecifics were used to form the shoal in each of the eight sociability test tanks and for each test day. Test trials were video‐recorded from above and subsequently tracked using custom tracking scripts in Python (version 2.7.5; www.python.org), providing detailed positional co‐ordinates for each individual during each boldness and sociability trial. To standardize hunger levels, individuals were fed three *Chironomus* sp. at the end of each day until all personality testing was finished.

To investigate the repeatability of behaviour, the key requirement of animal personality, individuals received two boldness sessions (on days 4 and 8 after individual housing) and two sociability sessions (on days 6 and 10). Based on the positional co‐ordinates during the personality trials, it was found that individual *G*. *aculeatus* spent mean ± s.e. 27·9 ± 1·4% of their time out of cover (range: 0·0–62·8%) during the boldness test and were at mean ± s.e. 47·9 ± 2·3 mm from the compartment housing conspecifics (range: 13·0–116·0 mm) during the sociability test. As individual *G*. *aculeatus* (*n* = 96) were repeatable in the time they spent out of cover (*r*_s_ = 0·41, *P* < 0·001) and in their average distance from the shoal compartment (*r*_s_ = 0·50, *P* < 0·001), boldness and sociability scores were calculated for each individual by averaging their behaviour across the two test sessions for each personality trait. Boldness was not correlated with sociability (*r*_s_ = 0·00, *P* > 0·05) and neither personality trait correlated with *L*_S_ (*r*_s_ = 0·11, *P* > 0·05; *r*_s_ = 0·10, *P* > 0·05, respectively).

A week after personality testing, during which two *G*. *aculeatus* had died from unknown causes, all individuals (*n* = 94) received a single *Chironomus* sp. daily for three consecutive days to minimize stomach fullness and to ensure that *Chironomus* sp. would be consumed immediately when provided (Beukema, 1968). Starting at 1430 hours on the fourth day of food restriction, individuals' maximum food intake was measured by dropping five medium‐sized *Chironomus* sp. (mean ± s.e. = 12·7 ± 0·4 mg wet mass, *n* = 50 worms) onto the feeding dish in each individual's home compartment. After 15 min, the number of *Chironomus* sp. eaten was determined and five additional *Chironomus* sp. were provided in the same manner unless some *Chironomus* sp. remained uneaten. In the latter case, no additional *Chironomus* sp. were provided during that round. If during a later round all *Chironomus* sp. were eaten, an additional five were provided. Individuals were considered satiated if they did not consume any *Chironomus* sp. for at least 30 min while *Chironomus* sp. were still available on their feeding dish. As the maximum daily food intake may be influenced by the speed at which *G*. *aculeatus* can empty their stomach, provisioning rounds were stopped after 3 h when all individuals were satiated. A generalized linear model (GLM) was run with *L*_S_, boldness and sociability as fixed factors to investigate how these variables affected the total number of *Chironomus* sp. eaten. The data were fitted to a Poisson error distribution with log‐link function, as appropriate for count data, and residuals were visually inspected to ensure homogeneity of variance, normality of error and linearity.

The maximum number of *Chironomus* sp. eaten during the feeding experiment varied considerably among individuals, ranging from 15 to 59 bloodworms (mean ± s.e. = 36·1 ± 1·1). *L*_S_ was the strongest predictor of food intake, with larger individuals eating significantly more *Chironomus* sp. [*P* < 0·001; Table I and Fig. 1(a)], although relative food intake in terms of percentage body mass dropped with *M* (*r*_s_ = −0·43, *P* < 0·001). These findings were unsurprising as larger individuals have larger stomachs and can thus consume more food, and are in line with the common finding that across teleosts a larger body mass is linked to a higher overall RMR but lower mass‐specific RMR (Clarke & Johnston, 1999). Next to *L*_S_, boldness was also positively correlated with maximum food intake [*P* < 0·01; Table I and Fig. 1(b)]. Keeping *L*_S_ constant at the average *L*_S_ (40·7 mm), the shyest and boldest individuals were predicted to still vary up to 20% in their food intake [32·2 and 40·1 *Chironomus* sp., respectively; Fig. 1(a)]. This shows that individuals with different personality types differ in their food intake even when inactive, *i*.*e*. not engaging in energetically expensive activities (Biro & Stamps, 2010) and when foraging is not directly linked to risk‐reward trade‐offs (Ioannou *et al.*, 2008). This complements existing evidence that bolder individuals tend to have higher feeding rates (Biro & Stamps, 2008), but is the first time this relationship has been shown for individuals at rest.

**Table I:** Coefficients of GLM on the maximum number of Chironomus sp. eaten by food‐deprived Gasterosteus aculeatus

	Estimate	s.e.	Wald statistic (*χ* ^2^)	*P*
*L*_S_ (mm)	0·05	0·00	93·03	<0·001
Boldness	0·35	0·13	7·31	<0·01
Sociability	0·00	0·00	0·00	>0·05

Data were fitted to a Poisson distribution with log‐link function (*n* = 94). Backward stepwise elimination was used and statistics for non‐significant terms were obtained by adding the non‐significant term to the minimal model. *L*_S_, standard length.

**Figure 1:**
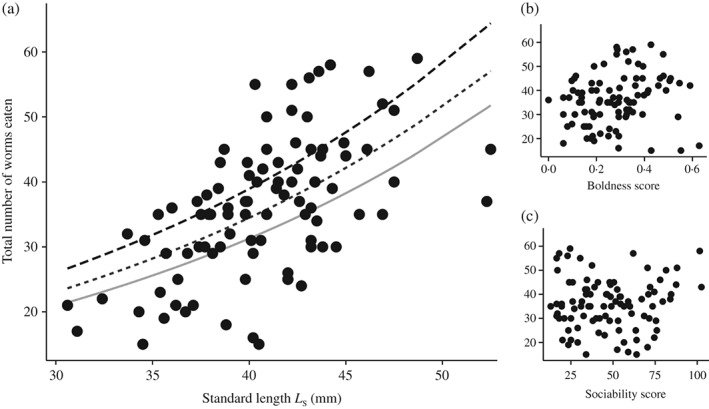
Scatterplots showing the relationship between (a) standard length (L_S_), (b) boldness (the average proportion of time out of cover during the risk‐taking test) and (c) sociability (the average distance from the compartment housing conspecifics in the sociability test) and the total number of Chironomus sp. eaten (n = 94). Lines in plot (a) are predicted maximum food intake for the shyest (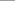), intermediate (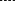) and boldest individuals (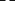).

Various mechanisms may explain why even the food intake of *G*. *aculeatus* that were inactive was positively linked to their boldness. First of all, bolder individuals may have relatively larger stomachs than shyer *G*. *aculeatus* and are therefore able to eat for longer. Secondly, bolder individuals may have a stronger motivation to eat, with shyer individuals not continuing to feed to the same fullness level. Thirdly, bolder individuals may be able to eat more due to a faster metabolism and digestion of food in their stomach, therefore enabling them to empty part of their stomach more quickly. Rapid digestion may especially be expected as the individual *G*. *aculeatus* had minimal stomach contents at the start of the experiment. All these explanations fit the ‘performance model’ (Careau *et al.*, 2008; Careau & Garland, 2012) and pace‐of‐life theory (Réale *et al.*, 2010), which suggests that an active, risky lifestyle is associated with well‐developed machinery for acquiring and processing food (Biro & Stamps, 2010), supporting the idea that boldness is linked to life‐history strategies (Stamps, 2007; Wolf *et al.*, 2007). These results are in line with the finding that bolder individuals have higher metabolic rates (Huntingford *et al.*, 2010), and that individuals with higher metabolic rates show increased risk‐taking after food deprivation (Killen *et al.*, 2011), as a larger ‘metabolic engine’ may come with higher maintenance costs (Biro & Stamps, 2010). Bold compared to shy individuals were not simply more motivated to feed because of having a larger *L*_S_, as the two were uncorrelated in this study, in line with other studies on *G*. *aculeatus* (Bell & Sih, 2007; Jolles *et al.*, 2015). As bolder individuals are more likely to consume prey in a risky environment (Ioannou *et al.*, 2008), and foraging fishes are less able to detect predators and predators more likely to target foraging prey (Krause & Godin, 1996), it may be suggested that risk is an important factor in the finding that bolder individuals had higher maximum food intake. This possibility is not likely however, as food was provided on a feeding dish at the plant cover, thus enabling individuals to eat while remaining concealed under cover. Furthermore, individuals were inactive and tested in their small home compartment after 3 weeks of acclimation time. Also, *G*. *aculeatus* were given 30 min to finish a batch of *Chironomus* sp. despite being able to finish it within seconds after provisioning (J. W. Jolles, pers. obs.). Future work could examine the link between boldness repeatability and metabolism in more detail by assessing metabolite concentrations in the water of individually housed fishes (Killen *et al.*, 2011, 2012), and investigate the possibility that shyer individuals may compensate for lower food intake by showing reduced activity.

In contrast to boldness, sociability was not linked to maximum food intake [*χ* ^2^ = 0·00; *P* > 0·05; Table I and Fig. 1(c)]. This result was predicted, as sociability is a personality trait that is not directly linked to energy production or metabolism. Nevertheless, it is likely that sociability has important indirect links to energy requirements. For example, more sociable individuals may have higher hydrodynamic benefits (Herskin & Steffensen, 1998) related to their spatial positioning in moving shoals (Jolles *et al.*, 2015), but may also have higher energy needs due to lower potential likelihood to discover food patches as well as scramble competition. This study is one of the first to test for an association between sociability and energetics (Careau & Garland, 2012). Future work is required to further investigate the link between sociability and energetics (Réale *et al.*, 2010), which may help to better understand the adaptive significance of sociability variation.

The results presented here on the feeding rates of food‐deprived *G*. *aculeatus* may be helpful for future fish studies that are focused on foraging dynamics or aim to use food reward paradigms, as they show that adult *G*. *aculeatus* are capable of eating up to 36 *Chironomus* sp. on average, or 0·46 g in wet mass, in a relatively short time scale (*c*. 1–3 h). Although no direct mass measurements were available, based on a large number of *L*_T_ and *M* estimates of *G*. *aculeatus* it was calculated that individuals ate roughly 32·0% of their body mass. This is very high considering that in the wild the average daily food intake rates of *G*. *aculeatus* have been shown to range between *c*. 1·5 and 16·9% of their body mass (Beukema, 1968; Manzer, 1976; Rajasilta, 1980; Allen & Wootton, 1984). This may for a large part be explained by the high wet mass of the food, *c*. 80% for Chironomidae (Armitage *et al.*, 2012). Ultimately, food intake is limited by the capacity of a well‐filled stomach, predicted to equal *c*. 5·5% of body mass (Beukema, 1968), and digestion rate, up to 15% stomach contents h^−1^ (Rajasilta, 1980). These results highlight that researchers studying personality traits and planning to use food rewards, such as for investigating the stability of personality or learning effects, should take into account that shy and bold fishes show intrinsic feeding differences irrespective of their body size.

In conclusion, individual *G*. *aculeatus* at rest varied considerably in their maximum food intake, even after accounting for body size. This variability correlated positively with boldness but not with sociability, as predicted by individual differences in life‐history strategies and growth–mortality trade‐offs associated with these personality traits.

We acknowledge funding from the Biotechnology and Biological Sciences Research Council (Graduate Research Fellowship to J.W.J) and the Association for the Study of Animal Behaviour (Research Grant to N.J.B).
